# Case report: Down syndrome regression disorder, catatonia, and psychiatric and immunomodulatory interventions

**DOI:** 10.3389/fpsyt.2024.1416736

**Published:** 2024-07-26

**Authors:** Michael H. Connors, Perminder S. Sachdev, James G. Colebatch, Mark S. Taylor, Julian Trollor, Adith Mohan

**Affiliations:** ^1^ Centre for Healthy Brain Ageing, UNSW Sydney, Sydney, NSW, Australia; ^2^ Neuropsychiatric Institute, Prince of Wales Hospital, Sydney, NSW, Australia; ^3^ Neuroscience Research Australia, UNSW Sydney, Sydney, NSW, Australia; ^4^ Department of Neurology, Prince of Wales Hospital, Sydney, NSW, Australia; ^5^ School of Clinical Medicine, UNSW Sydney, Sydney, NSW, Australia; ^6^ Department of Clinical Immunology, Prince of Wales Hospital, Sydney, NSW, Australia; ^7^ National Centre of Excellence in Intellectual Disability Health, UNSW Medicine & Health, UNSW Sydney, Sydney, NSW, Australia

**Keywords:** down syndrome, down syndrome regression disorder, electroconvulsive therapy, intellectual disability, immunomodulatory treatment

## Abstract

Down syndrome regression disorder (DSRD) is a rare condition involving subacute cognitive decline, loss of previously acquired developmental skills, and prominent neuropsychiatric symptoms, particularly catatonia, in people with Down syndrome. It is thought to involve both autoimmune and neuropsychiatric mechanisms. Research, however, is largely restricted to case studies and retrospective case series and is particularly limited in terms of prospective longitudinal follow-up. We report a case study of a person with DSRD who received both immunomodulatory (intravenous immunoglobulin; IVIG) and psychiatric interventions (electroconvulsive therapy, ECT) over two years with regular assessments using caregiver and clinician ratings. This revealed a small, unsustained response to IVIG and a rapid, sustained response once ECT was introduced. The case highlights the importance of multimodal assessment involving multiple medical specialties, the need to trial different therapies due to the condition’s complexity, and the significant barriers that patients and their families face in accessing care.

## 1 Introduction

Down syndrome regression disorder (DSRD) is a condition involving subacute cognitive decline, loss of previously acquired developmental skills, and prominent neuropsychiatric symptoms, particularly catatonia, in people with Down syndrome (1–4). While the condition has been known for some time (5, 6), limited research forces clinicians to be guided largely by case reports and retrospective case series (1–4, 7). Available research indicates that patients with DSRD have higher rates of autoimmune conditions than other people with Down syndrome (3) and that some patients improve with immunomodulatory therapy, such as intravenous immunoglobulin (IVIG) (3, 8–10). Features such as catatonia, developmental regression, altered mental state, and neurodiagnostic abnormalities may suggest greater likelihood of response to immunomodulatory therapy (3, 11, 12). Other research has found that patients with DSRD improve with psychiatric interventions, including antidepressants, benzodiazepines, and electroconvulsive therapy (ECT) (3, 10, 13). Altogether, these findings suggest both immunological and neuropsychiatric etiologies (3, 4), with a corresponding need to trial disparate treatment modalities for which there is limited prospective longitudinal research (11, 12). We report a case study of a person with DSRD who received immunomodulatory and psychiatric interventions over the course of two years with regular assessments using caregiver and clinician ratings. The case extends previous research by assessing both types of intervention prospectively using fine-grained measures, whilst highlighting practical challenges faced by clinicians, patients, and families during treatment.

## 2 Case description

### 2.1 Background

Anna (pseudonym) was a woman in her late teens with Down syndrome and a moderate intellectual disability who lived with her parents and attended supported education. She completed most activities of daily living with minimal support and initiated activities, such as walking her dog, vacuuming, and preparing for school. She could also use an iPad, make coffee independently, and bake with supervision. Testing of intellectual and functional abilities was conducted in middle childhood. On the Wechsler Intelligence Scale for Children-IV (14), subtest composite scores were in the extremely low range (scores 47–53) with a full scale intelligence quotient of 40. On the Adaptive Behavior Assessment System-3 (15), scores were in the extremely low range (composite scores 58–61) with the exception of the social domain, where she scored in the low-to-below average range (composite score 80). Her medical history was limited to coeliac disease and several minor surgeries, with no previous psychiatric concerns.

Anna presented with her parents to a neuropsychiatric clinic following six-to-nine-months of cognitive and functional decline and worsening catatonia. Her parents described noticing initially that Anna had appeared socially withdrawn, such that she refused to engage with her support workers and teachers, and lost interest in activities that she previously enjoyed, including movies, parties, and cooking. They reported a clear deterioration over two-to-three months in both her cognition, as evidenced by worsening spelling and recall of birthdates, and function, reflected in reduced self-care and activities. They also noticed that Anna had started talking to herself, often in apparent conversation with people who were not present. After four-to-five months, Anna’s parents reported observing Anna performing atypical movements, holding bizarre postures for long periods, remaining mute, and not responding to questions. Anna’s parents noted a gradual worsening of these symptoms, as well as increasing insomnia, over several months. They described Anna alternating between periods of immobility with periods of hyperactivity. Anna’s parents sought opinions from many medical specialists and joined parent advocacy groups before they were referred for neuropsychiatric assessment at our center.

Across reviews, Anna demonstrated catatonic features, including selective mutism, posturing, negativism, grimacing, and stereotypies. No psychosocial precipitant was identified to explain Anna’s cognitive and behavioral changes. The consensus impression of multiple specialists was that Anna met criteria for probable DSRD according to international guidelines (1).

### 2.2 Investigations

Extensive investigations, as recommended by international consensus guidelines (1), were similarly unable to identify a cause. Serum investigations were notable only for positive thyroglobulin antibodies (8.7 IU/mL, normal < 4.0 IU/mL) with normal thyroid function. Serum limbic encephalitis antibodies, anti-neuronal antibodies, double stranded DNA antibodies, and antibodies to extractable nuclear antigens were not detected. Cerebrospinal fluid (CSF) studies demonstrated an acellular CSF with normal protein and neopterin concentrations, oligoclonal bands not detected by isoelectric focusing, and limbic encephalitis and anti-neuronal antibodies not detected by indirect immunofluorescence. Electroencephalogram and brain scans using magnetic resonance imaging and positron emission tomography were unremarkable.

### 2.3 Differential diagnosis

An important differential diagnosis was schizophrenia. In addition to catatonia, which can occur in schizophrenia, Anna started talking to herself, a possible sign of hallucinations, and her functional decline could reflect schizophrenia’s prodrome. Schizophrenia also has an elevated prevalence in Down syndrome (16). While this diagnosis could not be excluded, DSRD was considered more likely given the acute-onset and marked cognitive decline and rapid developmental regression that is unusual for schizophrenia. Catatonia can also occur in Down syndrome, including in the absence of DSRD (17, 18). Self-talking is likewise common in Down syndrome and does not necessarily indicate the presence of psychosis (19). In Anna’s case, her self-talking occurred only occasionally for short durations and no other definite signs of psychosis were noted, suggesting that it might represent stereotyped and repetitive language. These features indicated that she did not meet diagnostic criteria for schizophrenia (20) during the assessment and follow-up period, making this diagnosis less likely. This would need to be reconsidered if she were to develop further features of schizophrenia in the future.

Another differential diagnosis was major depressive disorder with psychotic features. This could explain Anna’s social withdrawal, loss of interest in activities, insomnia, and functional decline (20). Cognitive impairment, catatonia, and psychosis can also occur in severe depression (20, 21). Depression likewise has an elevated prevalence in Down syndrome (16). Developmental regression, however, is very atypical in depression and the severity of her cognitive decline is unusual. Anna also did not report low mood to her parents, who did not consider her to be depressed. Such issues led our team to consider DSRD to be a more likely diagnosis, though with the possibility of depression being present as a comorbidity or contributing factor.

### 2.4 Treatment

Treatment was planned based on existing evidence and involved specialists in neuropsychiatry, neurology, clinical immunology, and intellectual disability psychiatry. Anna was reviewed regularly over the following year. This involved two key measures in addition to her parents’ qualitative reports. First, the Bush-Francis Catatonia Rating Scale (22) was completed by a clinician to assess catatonia symptoms. Second, Anna’s mother was asked to provide a single global rating of Anna’s overall level of functioning and symptoms as a percentage ranging from Anna’s worst (0%) to her premorbid baseline (100%), with further prompts about ratings provided on previous visits. For this latter measure, the first two ratings were obtained retrospectively; all other ratings were obtained contemporaneously.

Anna was initially trialed on fluoxetine (up to 30 mg) and high dose oral lorazepam (up to 9 mg daily), with negligible change in her symptoms after two months. While higher doses of lorazepam have been used in select cases (23), most studies of lorazepam as a treatment of catatonia have used doses of 9 mg daily or less (24) and no studies have trialed higher doses in catatonia associated with DSRD. A further consideration was patient tolerance with greater risk of adverse effects with higher doses, particularly in neurodevelopmental disorders (25). This failure to respond to lorazepam does not exclude a diagnosis of catatonia as studies have shown a wide range of response rates (23).

Given this lack of response, ECT and immunomodulatory therapies were considered based on available research suggesting efficacy of both in DSRD (3, 8–10), as well potential risks and side-effects. The options were discussed with Anna’s parents, who expressed a strong preference for immunomodulatory treatment. Immunomodulatory treatment was then commenced one month after our initial review. This involved intravenous immunoglobulin (IVIG; 1g/kg body weight) infusions four-weekly with concurrent dexamethasone (4 mg three times daily for 3 days, tapered to 4 mg daily for a week after the first treatment, though without tapering on subsequent treatments).

### 2.5 Outcome and follow-up

**Figure 1:**
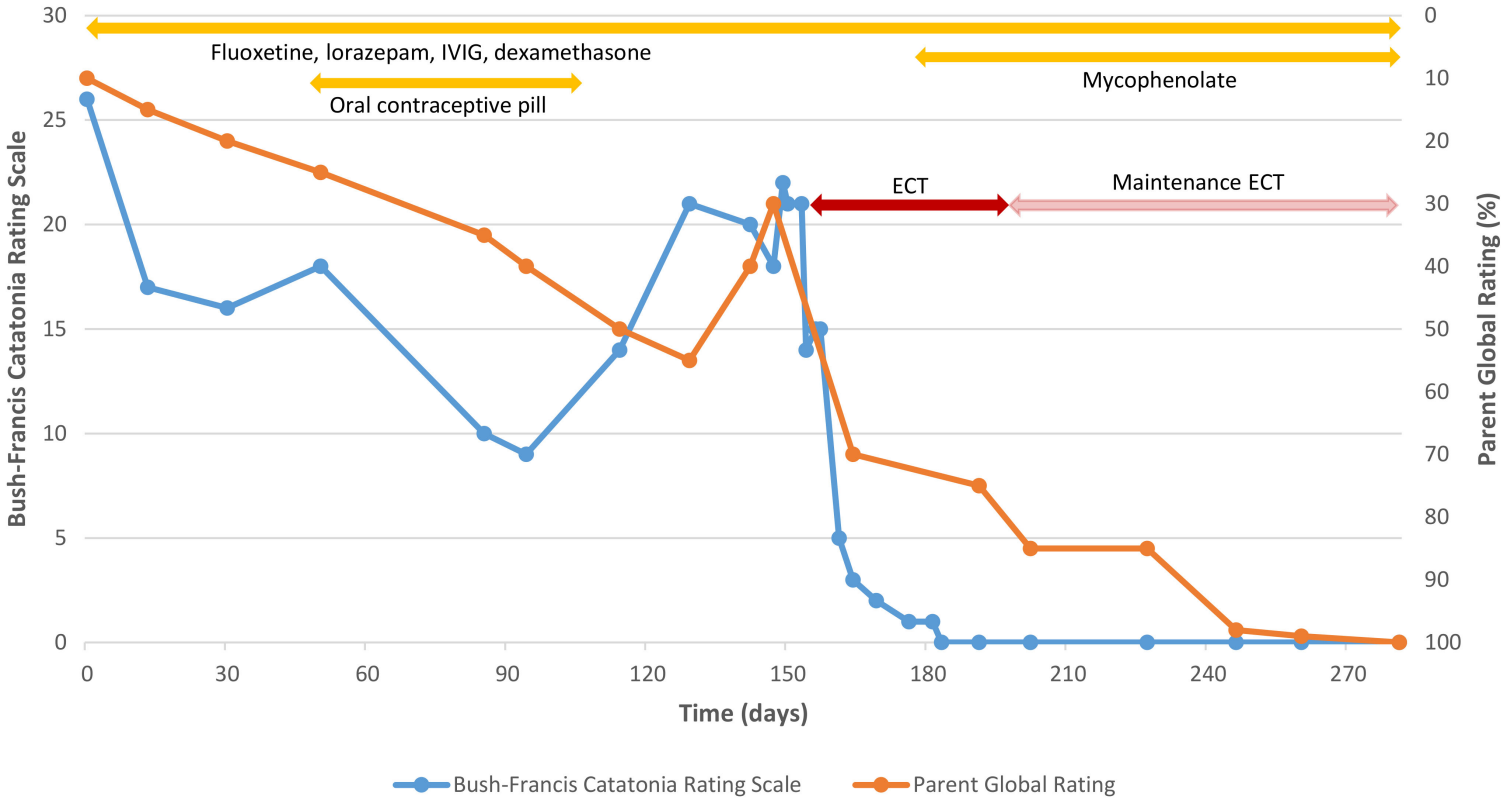
Clinician ratings of catatonia and parental ratings of global functioning over time. Catatonia rating scales were completed by a clinician using the Bush-Francis Catatonia Rating Scale. Parent global ratings of overall function and symptoms were completed by the patient’s mother on a scale from 0% (worst) to 100% (premorbid baseline). The figure depicts the first nine months of treatment. Ratings remained at baseline over the following three months with ongoing maintenance ECT and other regular treatments (fluoxetine, lorazepam, and monthly IVIG and dexamethasone, but not mycophenolate, which was ceased five months after starting).

Anna’s parents reported resolution of her insomnia and gradual improvements in social engagement and cognitive skills over the following three months (improving around 10% of premorbid functioning each month in parental ratings), enabling Anna to return to school. During clinical reviews, Anna demonstrated gradual, albeit fluctuating, improvements in catatonia (**Figure 1**). She was started on an oral contraceptive pill in preparation for immunosuppressive therapy but ceased this after several weeks due to menorrhagia and dysmenorrhea.

Three months after starting IVIG treatment, Anna’s parents reported a further worsening of Anna’s catatonia. This matched a deterioration in catatonia ratings captured in clinic (**Figure 1**). Her parents nevertheless indicated that, despite this, Anna’s functioning and social engagement continued to improve when she was not catatonic (reaching 55% of premorbid functioning in parental ratings). After another month, however, they reported that Anna’s functioning and social engagement also declined (dropping to 30% of premorbid functioning). In addition, they reported that Anna had started speaking to herself more frequently and exhibiting urinary and fecal incontinence despite being toilet trained.

Given this decline, Anna was started on a course of ECT. Organizing this posed logistical challenges due to the lack of established clinical pathways for people with intellectual disability and Anna’s transition from pediatric to adult services. Anna was admitted to a neurology ward to avoid exposing her to the unpredictable environment of an adult psychiatric ward. ECT was approved by a tribunal under the state’s Mental Health Act because the state does not accept guardian consent for ECT.

ECT was administered with right unilateral lead placement and brief pulse width (1.0 ms) at five times the seizure threshold, initially three times weekly. While bilateral ECT has traditionally been recommended for treatment of catatonia (26), there has been very limited systematic study of electrode placement in this context (27) and other electrode placements have been found effective (28). We decided to trial right unilateral lead placement initially given its lower risk of cognitive side-effects (29), which we considered an important consideration given Anna’s established intellectual disability, the fact that eating and drinking were not compromised, and evidence of efficacy in case series (24, 28).

In response to ECT, Anna showed dramatic improvements (**Figure 1**). Her catatonic symptoms, present for a year, rapidly subsided and she became much more socially interactive, speaking spontaneously with clinicians for the first time. Anna’s parents noted resolution of her self-talking and incontinence, as well as marked improvements in her cognition and functioning, including in vocabulary, memory for birthdays, and participation in activities, such as using an iPad, vacuuming, cooking, and drama classes (reaching 85% of premorbid functioning in parental ratings).

After 12 treatments, Anna’s ECT was shifted to a maintenance phase, with ECT administered at gradually greater intervals of up to six weeks. Over the next three months, Anna’s parents reported further improvements, such that they considered Anna to be at baseline. Mycophenolate was commenced after the initial response to ECT as a steroid-sparing immunomodulatory agent for longer-term treatment, though was ceased after five months due to side-effects, namely abdominal pain. Over a further three months with maintenance ECT and other regular treatments, Anna remained at baseline according to both her parents and clinician ratings of catatonia (not shown in **Figure 1**).

After this first year of treatment, ECT was ceased for four months, while IVIG continued. She also received a dose of rituximab (3). At this point, Anna gradually started to have brief, intermittent episodes of freezing, muteness, and aggression, though without signs of cognitive or functional decline or persistent catatonia that was evident in clinical review (Bush Francis Catatonia Rating Scale scores in clinic remained < 3; parental ratings were not continued given the episodic nature of concerns and challenges establishing a consistent baseline). A further course of 12 ECT treatments using the previous protocol was then initiated over five months, leading to the cessation of these episodes. IVIG was ceased due to inadequate evidence of a clear and sustained response. ECT was then also ceased for a further five months without any re-emergence of persistent cognitive or functional decline or catatonia, though with a later gradual return of brief, episodic aggression (e.g., striking caregivers, then apologizing immediately and requesting a return to hospital for treatment). Low dose antipsychotic medication (aripiprazole up to 10 mg) was recently commenced with a plan for behavioural interventions targeting this specific symptom.

### 2.6 Ethics statement

Anna’s mother provided written informed consent for treatment and publication of this case report given that Anna did not have capacity to do so due to her intellectual disability. Both parents read and approved the manuscript of this case report for publication. The local ethics committee granted an ethics waiver as all treatments provided and all information collected were part of routine clinical care with appropriate precautions around confidentiality and consent.

## 3 Discussion

In longitudinal follow-up, Anna showed a small, positive but fluctuating response to IVIG that was not sustained beyond three months. By contrast, Anna showed a rapid response to ECT, leading to a resolution of symptoms that was sustained for more than six months with maintenance ECT. The effectiveness of ECT provides further support for its use in select patients. While there are challenges in generalizing the findings given the single case study design, the findings suggest that ECT could be considered earlier, particularly when catatonia is prominent. The findings are consistent with other research suggesting that ECT may be effective for DSRD (3, 10, 30) and catatonia in Down syndrome (31). The findings are also consistent with retrospective research suggesting that a lack of neurodiagnostic abnormalities may predict responsiveness to ECT and a lack of responsiveness to IVIG (3, 11). Anna’s case extends previous reports by assessing responses to both treatments prospectively using clinician and parent ratings and clearly demonstrating a marked, contiguous relationship between ECT and improvements on both measures.

By contrast, the equivocal long-term response to IVIG, in combination with the absence of definitive biomarkers, raises questions about the role of immunological mechanisms in Anna’s case, but cannot exclude them. Anna’s response is also consistent with recent research suggesting that most patients with DSRD improve initially with IVIG, though this may not be sustained, and that improvements in catatonia can be associated with the worsening of other neuropsychiatric symptoms (12). Such issues highlight the challenges in managing a heterogeneous condition in which immunological factors contribute to a proportion. Of note, ECT appears to be effective in organic catatonia, though possibly to a lesser extent than functional catatonia (26, 27), so is likewise not necessarily informative about etiology. It is also possible that ECT may have immunomodulatory properties (32). Given such uncertainty about underlying mechanisms and the risks associated with many immunomodulatory treatments, there remain ongoing questions about prognosis and optimal long-term therapy, including the role of further ECT without IVIG. There is also uncertainty about the potential role of immunomodulatory treatments in managing catatonia associated with other conditions, particularly in cases where immunological factors are suspected but not definitively established.

Anna’s case reveals further challenges in assessing treatment response. Due to limited research, clinicians need to make assessments of effectiveness with some uncertainty about the evidence base. Other challenges include communication barriers with people affected by both intellectual disability and catatonia, expected variability in symptoms, and potential confounds, such as the frequent need to pragmatically trial concurrent interventions. In Anna’s case, while there was concordance between clinician rating of catatonia and parents’ ratings of global functioning overall, significant divergences still occurred. In particular, her parents described a near continuous improvement in functioning despite the re-emergence of worsening catatonia. With time, however, her functional ratings also declined, ultimately necessitating ECT. While the overall response to ECT was clear, such divergences indicate limitations in the measures.

Both the clinician and parent-rated measures used have specific shortcomings. Clinician ratings of catatonia assess only this behavioral abnormality at single time-points and do not capture other domains of function or behavior over time. By contrast, parental ratings of global functioning require parents to integrate diverse domains, each subject to variability, over longer periods of time, so could be vulnerable to recall and other biases. Parental ratings of global functioning are also difficult to complete for episodic symptoms. While clinician and parental ratings largely corresponded, the discrepancies that occurred, as well as the variability of individual ratings, indicate the challenges in assessing progress and the need to consider multiple indicators. A further limitation of the study was the fact that standardized measures of function were not used, which can be more difficult to administer over short time periods with frequent follow-up. Other measures that could be used include the Neuropsychiatric Inventory, a timed 25-foot walk, and the Clinical Global Impression Scale (11, 12). Standardizing protocols for assessment may assist in evaluating interventions and conducting more rigorous clinical trials across multiple sites.

Anna’s case highlights further issues related to the challenges that people with DSRD and their families have in accessing treatment. Anna’s mother actively sought out many specialists and parent groups before being reviewed at our center, with additional logistical difficulties arising from the transition between pediatric and adult services. The lack of established clinical pathways for people with intellectual disability, in combination with DSRD’s complexity and need for coordinated input from multiple clinical specialties, pose particular barriers. Patients with other rare disorders involving intellectual disability and neuropsychiatric manifestations also face similar obstacles (33). We addressed the challenges of Anna’s case by working as an interdisciplinary team with regular follow-up. In future, the development of more formalized clinical pathways, possibly led by existing intellectual disability services, might help to ensure that people with such conditions and their families are able to access appropriate care.

## 4 Patient perspective

An account by Anna’s mother:

*Anna lived the funnest life, and was sassy, funny, loving, independent, and healthy. This changed when, in her late teens, she become increasingly withdrawn, combative, and mute. She developed severe insomnia and started losing her ability to communicate and her independence. At times, she would become locked in, ‘frozen’, immovable. At others, she would run around in circles, whispering, lost to her own world.*

*It is impossible to describe the devastation of what this was like to witness. For months I sought answers. I contacted many doctors, most of whom had never come across these symptoms or dismissed it as ‘just Down syndrome.’ Desperate, I searched literature and forums and discovered that Anna’s symptoms were almost identical to a small group of young adults with Down syndrome around the world - and that most were being similarly dismissed.*

*As my health, work, family and social relationships broke down, I started to find answers. I located a pediatric neurologist who recognized the symptoms for what they were and immediately referred Anna for assessment and treatment. This breakthrough was short-lived. Anna was no longer eligible for care within the pediatric system. She would need to pivot to adult care and we would have to start again.*

*Anna began to deteriorate rapidly. I was forced to choose between Anna and her siblings, Anna and work, Anna and outside life. As her advocate, I again had to fight to convince specialists, this time in the adult healthcare system, that I was to be believed, that her symptoms were genuine, that this was not ‘Anna’ or ‘Down syndrome’, that Anna was worthy of care equal to anyone who walked through the door.*

*We were lucky. We eventually found a team who worked tirelessly and collaboratively towards her recovery, treating her with care and respect. They have trusted and respected me as Anna’s advocate, recognizing the value of greater ‘carer’ consultancy. As a result, Anna is back. Sassy, funny, happy, thriving and ready to live her very best life.*

## Data availability statement

The original contributions presented in the study are included in the article. Further inquiries can be directed to the corresponding author.

## Ethics statement

The requirement of ethical approval was waived by the South Eastern Sydney Local Health District (SESLHD) Human Research Ethics Committee for the study because all treatments provided and all information collected were part of routine clinical care with appropriate precautions around confidentiality and consent. The study was conducted in accordance with local legislation and institutional requirements. As the participant did not have capacity to provide consent, written informed consent for participation in this study was provided by the participant’s legal guardians/next of kin. Written informed consent was obtained from the participant’s legal guardian/next of kin for the publication of any potentially identifiable data included in this article.

## Author contributions

MC: Conceptualization, Data curation, Investigation, Methodology, Project administration, Writing – original draft, Writing – review & editing. PS: Conceptualization, Methodology, Supervision, Writing – review & editing. JC: Conceptualization, Methodology, Supervision, Writing – review & editing. MT: Conceptualization, Methodology, Supervision, Writing – review & editing. JT: Conceptualization, Methodology, Supervision, Writing – review & editing. AM: Conceptualization, Methodology, Supervision, Writing – review & editing.
